# Influence of multiple spatiotemporal resolutions on the performance of urban growth simulation models

**DOI:** 10.1016/j.isci.2023.108540

**Published:** 2023-11-30

**Authors:** Tingting Xu, Heng Su, Biao He, Aohua Tian, Jianing Guo

**Affiliations:** 1 Chongqing University of Posts and Telecommunications, Chongqing, China; 2 Guangdong–Hong Kong-Macau Joint Laboratory for Smart Cities, Shenzhen, China; 3 Shenzhen University, Shenzhen, China

**Keywords:** Geography, Artificial intelligence, Machine learning, Urban planning

## Abstract

The study developed a framework to investigate the impact of multiple spatial and temporal resolutions on urban growth simulation. The research utilized the convolutional long short-term memory (ConvLSTM) model and three regular models and data from 2009 to 2017 to simulate the urban area of Liangjiang New District in 2018 and determine the optimal spatiotemporal resolution for urban expansion models. The results indicated that the ConvLSTM model has the best simulation result and the ideal temporal resolution for Liangjiang district is to include the previous two years of data, with an optimal spatial resolution of 90 m and a spatiotemporal simulation zone within a two-year time step and 100 × 100 spatial information filter. At this combination, the kappa value of the ConvLSTM is 0.87 which is about 5% higher than others. Our findings revealed that the characteristics of input data can have a significant impact on simulation results and should be carefully considered during the simulation process.

## Highlights

• We obtained optimal input data resolution in simulating fast-growing urban area
• We revealed the effects of different spatiotemporal combinations on urban models
• The best spatiotemporal zone is within past two years and 100 × 100 spatial filter
• We provide an optimization process for suitable spatiotemporal resolution selection

## Graphical abstract

**Figure:**
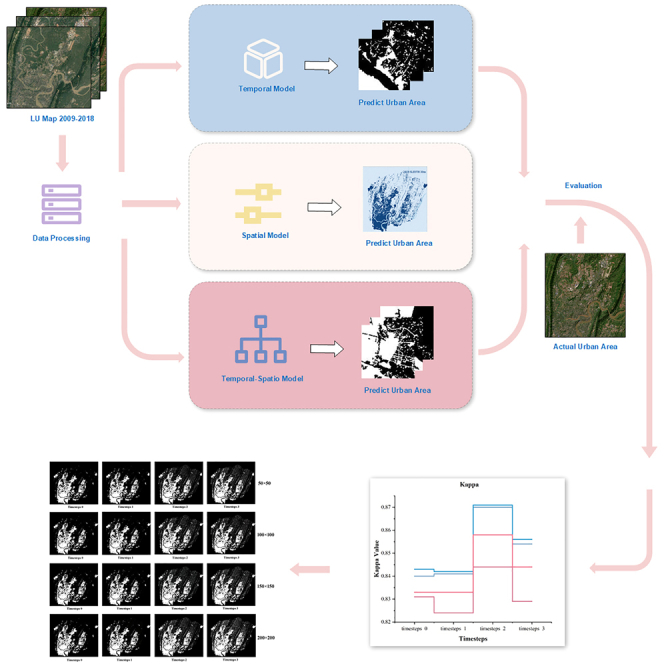

## Introduction

Predicting urban growth accurately can relieve the pressure on land and promote sustainable development of large cities, increasing the efficiency of urban land use.^1^ Urban expansion can be simulated with support vector machine (SVM),^2^ logistic regression, artificial neural network,^3^ and many other models. Cellular automata (CA) is also one of the prevailing methods^4^ with various versions, including variable improved cellular automata models such as SLEUTH, CA-Markov, and FLUS.^5^^,^^6^^,^^7^ These models simulate and predict urban expansion from historic land use and land cover data as the inputs to train and validate the model to predict future.^8^^,^^9^

Many urban growth simulations select a resolution that lack a clear theoretical basis and the impact of selected resolutions on the accuracy of urban expansion simulations has not been thoroughly explored modeling urban boundary expansion using 30 m resolution data. This observation is supported by Guo et al.^10^ and Chakraborti et al.^11^ Shafizadeh-Moghadam et al.^12^ also used 30 m spatial resolution to simulate the urban expansion. Meanwhile, they evaluated the spatiotemporal generalization capabilities of statistical and machine learning models to simulate land expansion in built-up areas.^13^ It has been demonstrated that different scales of study, which contain distinct spatial information, have a significant impact on urban expansion simulation, and the scale effect cannot be ignored because all inputs fitting into the model have their own scale of representation.^14^ While a fine resolution is preferred, it exponentially increases the computation cost. Conversely, a coarse resolution may neglect local and small-scale developments.

Accurate simulation of urban development is a complicated process that necessitates integrating various temporal and spatial data inputs. Among the important influential factors, the speed of urban expansion can have varying temporal resolutions. Current studies on urban expansion typically have a relatively coarse time step (usually 5–10 years) in rapidly urbanizing areas to efficiently obtain consecutive temporal information on urban development for urban economic and social planning. There is limited research on the impact of different temporal resolutions on the accuracy of urban expansion simulation. Kaewthani et al.^15^ developed a temporal consistency evaluation that yielded higher accuracy than that of without temporal consistency. Chai et al.^16^ applied spatial-temporal filtering in OCSVM, resulting in an accuracy increase of between 4.3% and 11.7%. Xu et al.^17^ used high temporal resolution data to simulate urban land use change by the long short-term memory (LSTM) model, yielding higher accuracy than MLP. Boulila et al.^18^ utilized a convolutional LSTM (ConvLSTM) network to forecast urban expansion patterns that produced a good classification accuracy of 92.19%. Despite the success of aforementioned studies, it is unclear whether high temporal resolution input data can improve urban development simulation.

To explore the impact of time on model simulation accuracy, researchers are considering the use of recurrent neural networks (RNNs), which have demonstrated exceptional ability in modeling sequential data and are widely used in various applications with excellent results.^19^ RNNs are particularly well suited for handling time series data, which is essential in modeling land use change and urban expansion. However, RNNs struggle to capture long-term dependencies, which means that for longer sequences of inputs, previous information may be lost, resulting in models that are unable to make accurate predictions. Additionally, the concatenation of gradients in the backpropagation process can cause the problem of gradient disappearance or explosion when the gradient is small or large, which can affect model stability and the learning rate. To address these issues, LSTM model is used which introduces a gating mechanism to control the flow of information through different parts of the network. This mechanism effectively addresses the long-term dependency problem of traditional RNNs, and it can also alleviate the problems of gradient disappearance and explosion, making the training of LSTM more stable. The study will use the LSTM model to conduct experiments on data containing different temporal information.

Different time series have unique characteristics,^20^ but many researchers have only focused on one-step-ahead predictions. Pan et al.^21^ studied the urban expansion of BTHUA during 1992–2015 by LSTM but the model could not learn the multi-step effect on urban expansion simulation. Liu et al.^22^ confirmed the validity of the model performance, but the effect of different time series combinations on model performance is not discussed in detail. These one-step-ahead predictions are not practical for urban expansion simulations as they do not show model sensitivity to different time steps inputs.

By assessing the influence of various spatial and temporal resolutions on the accuracy of urban expansion simulations, we aim to address the limitations of previous studies by combining different spatiotemporal resolutions of input data at the model level. This will be accomplished by employing the ConvLSTM model to investigate whether different spatial and temporal resolutions impact the urban expansion model. The ConvLSTM model enables us to simulate spatiotemporal information simultaneously.^23^ Moreover, the combination of time and space resulting from this approach will facilitate determining the optimal spatiotemporal resolution for simulating urban expansion at a specific scale. In this paper, we investigate such effect of the ConvLSTM model and other models, such as SLEUTH, IDRISI, and FLUS on urban expansion in Liangjiang New District, Chongqing, at various temporal and spatial resolutions. This research will overcome the deficiency of previous studies that did not train the model using spatiotemporal data of varying resolutions.

## Study area and data

### Study area

**Figure 1:**
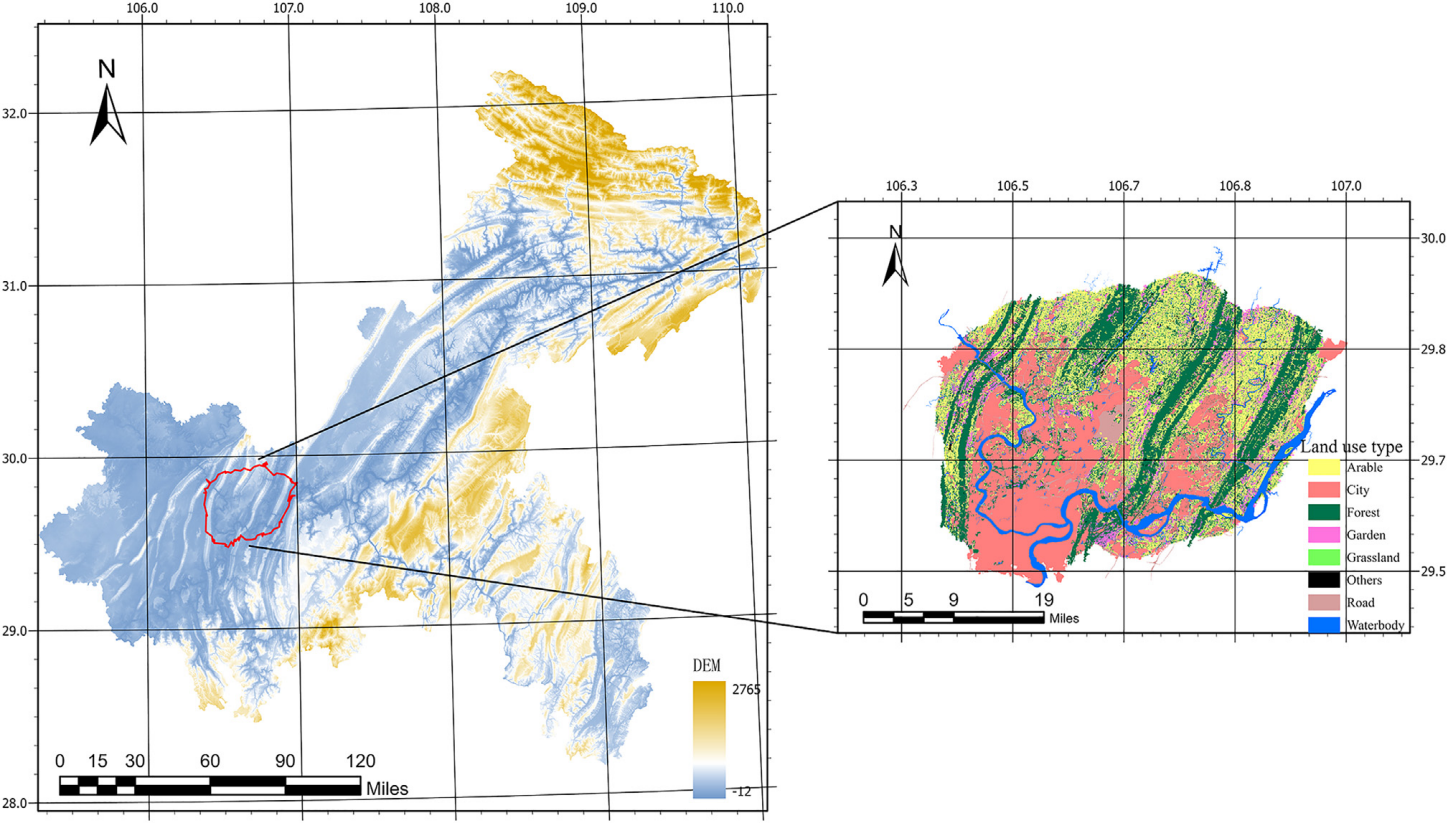
Study area: The Liangjiang New District of Chongqing, China

The study area is situated in the Liangjiang New District, Chongqing, which is one of the most prominent cities in Southwestern China (Figure 1). Located at the upper reaches of the Yangtze River, Chongqing is the largest municipal district in China with an area of over 82,400 km^2^.^24^ The mountainous terrain has created unique geographical advantages such as water conservation, tourism, and scenic environment. The district has far-reaching development plans and potential in the fields of economy, science and technology, and culture. As one of the fastest-growing areas in China, this study area provides an excellent simple to investigate how different spatial-temporal resolutions could affect the models in simulating urban expansion.

### Data

**Figure 2:**
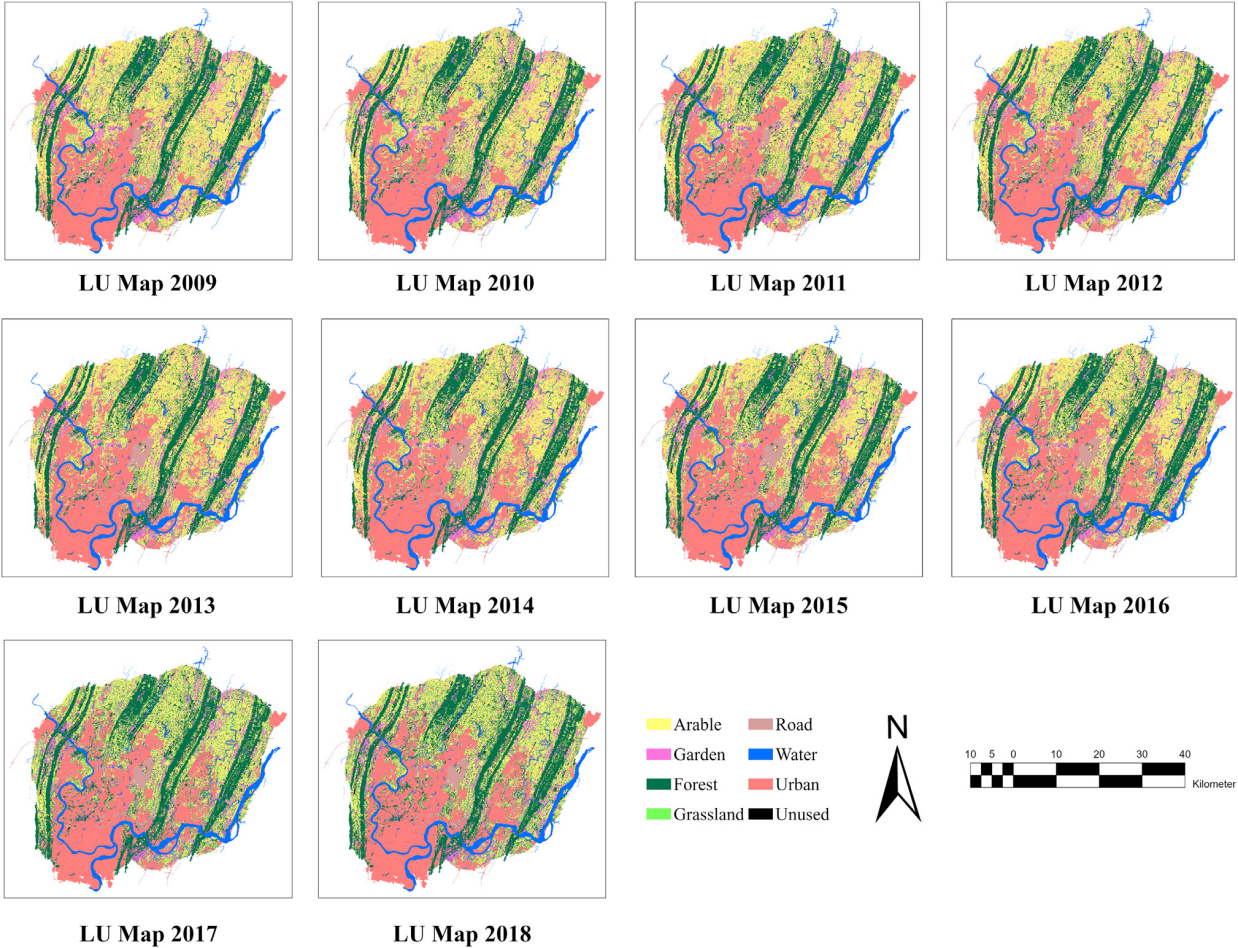
Time series land use map (2009–2018) of research area

Table 1 presents the utilized data which include land use data from 2009 to 2018 with a spatial resolution of 30 m (Figure 2), population data, and nighttime light data in 2010 and 2015. The 30 m resolution digital elevation model (DEM) data were employed to generate Slope and Hillshade. All land use data were acquired from Chinese Resource and Environment Science and Data Center (RESDC:https://www.resdc.cn/), including eight land use types (Arable, Garden, Forest, Grassland, Road, Water, Urban, Unused). These data were resampled into 90, 180, 450, and 900 m using the nearest-neighbor algorithm. Water bodies were extracted from the land use data as exclusion. In addition, to explore how different spatial resolution affects the model performance in simulating urban expansion, the urban area raster layer was aggregated to four grid sizes of 50 × 50, 100 × 100, 150 × 150, and 200 × 200. Also, four different time steps: 1, 2, 3, and 4 years were selected to incorporate previous temporal information in training the ConvLSTM model.

**Table 1:** Data information table

Data	Used model	Spatial Resolution	Year	Source
Land use	–	30 m	2009–2018	RESDC
Land use	SLEUTH, FLUS, IDIRIS	90, 180, 450, and 900 m	2009–2018	Resampled from land use data
Urban, Exclusion, Roads	SLEUTH, FLUS, IDIRIS	30, 90, 180, 450, and 900 m	2009–2018	Extract from land use data, OSM
DEM	SLEUTH, FLUS, IDIRIS	30 m	–	RESDC
Slope& Hillshade	SLEUTH, IDIRIS	30, 90, 180, 450, and 900 m	–	Derived by DEM
Nighttime & Population	FLUS, IDIRIS	30 m	2010 & 2015	RESDC
Urban Gary Image	ConvLSTM	30 m	2009–2018	Process by ArcGIS

**Figure 3:**
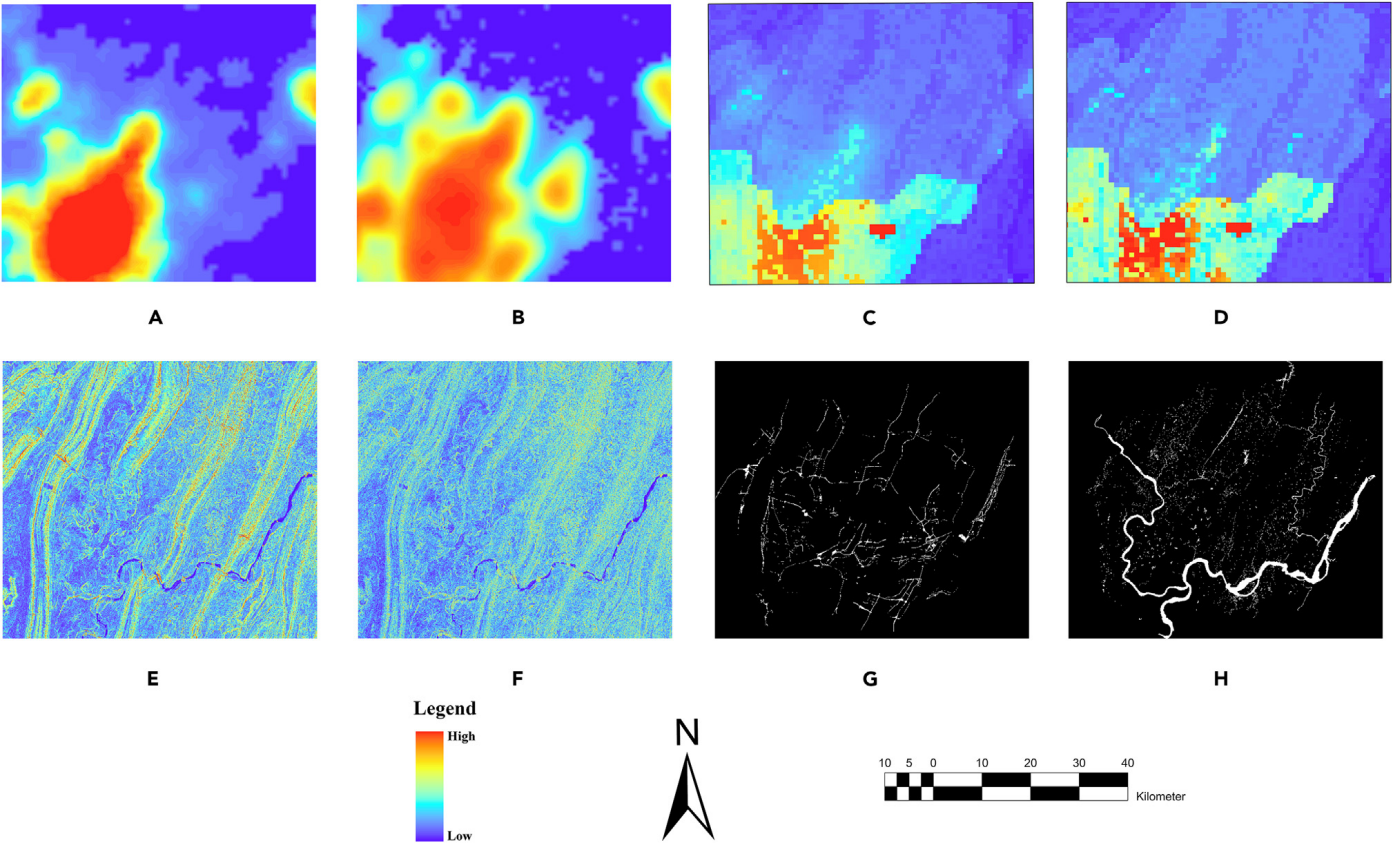
Driving factor images of the study area (A) Nighttime light (NTL) 2010, (B) Nighttime light (NTL) 2015, (C) Population 2010, (C) Population 2015, (E) DEM, (F) Slope, (G) Roads, (H) Exclusion.

Population growth, economic development, urban infrastructure development, and natural and environmental factors are the most important drivers of urban expansion.^25^ In this study, we used DEM, slope, and exclusion data to present the natural and environmental driving factors whereas nighttime lights and population data are the social factors (Figure 3). Moreover, roads are a crucial element of urban areas and play a significant role in determining the direction of urban development. Therefore, road data were also incorporated.

## Results

The urban area underwent substantial changes between 2009 and 2018 within study area, increasing from 547 to 750 km^2^. From 2009 to 2016, the urban area grew rapidly at a rate of 2.22% and continued to expand from 2016 to 2018, albeit at a slower rate of 1.03%. This complex urban change presented a considerable challenge for the model’s performance but was also well suited for examining the impact of different temporal and spatial resolutions on the model’s performance.

### Temporal effect

**Figure 4:**
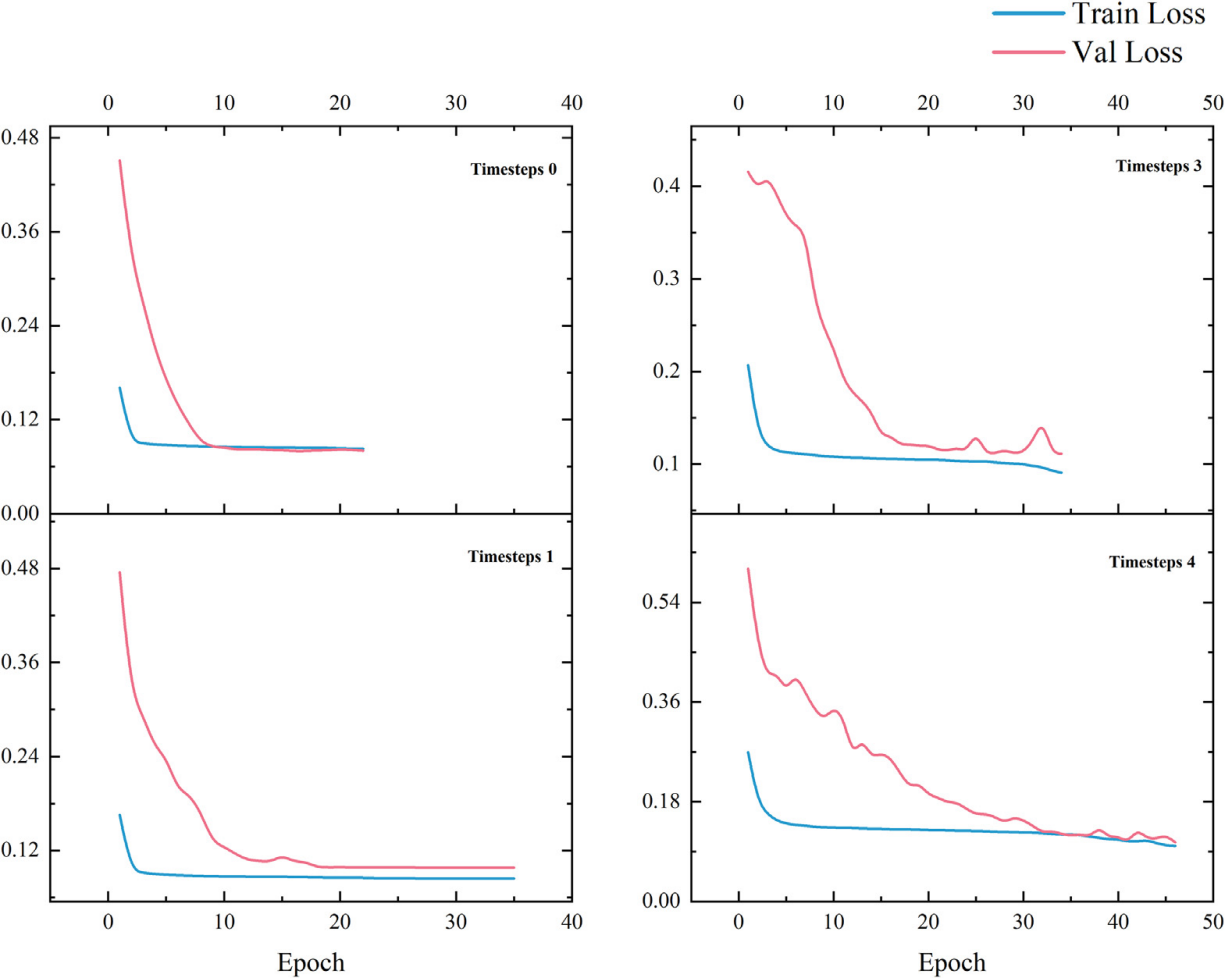
Temporal model training loss value curve

For the temporal evaluation, we trained different prediction models by segmenting the original image into 100 × 100 sub-images and processing them to contain data with different temporal resolutions. The model training loss results are shown in Figure 4.

It is noticeable that when the training data contain the same spatial information, the model trained without past time data has the lowest cross-entropy loss of 0.0804. The model trained with past one year data has the lowest cross-entropy loss of 0.0982, while the model trained with past two years data has the lowest cross-entropy loss of 0.1113. Lastly, the model trained with past four years data has the lowest cross-entropy loss of 0.1067.

In Table 2, we compared the predicted urban area of Liangjiang New District in 2018 by the model with the actual urban area in 2018 using Kappa coefficient and Fuzzy Kappa as accuracy criteria. The table shows the accuracies, and we observed that the inclusion of different temporal information resulted in differences in the results under the 100 × 100 spatial information range. Under timesteps 0 and timesteps 1, the models exhibited similar prediction performance, with Kappa coefficients of 0.843 and 0.842, respectively, and a Fuzzy Kappa of 0.884 for both. However, the models trained by including temporal data from the past two and three years showed improved prediction performance. The Kappa coefficient of timesteps 2 was 0.871, higher than timesteps 0 (0.843) and timesteps 1 (0.842), with a Fuzzy Kappa of 0.948, also higher than timesteps 0 and timesteps 1 (0.884). This indicates that a finer temporal resolution enhances the model’s predictive performance, and temporal resolution is a crucial factor that cannot be overlooked in urban expansion studies. The accuracy of the model trained with data containing three years of past time (timesteps 3) was similar to timesteps 2, but the prediction performance was inferior, with a Kappa coefficient of 0.856 and Fuzzy Kappa of 0.936. These results confirm that different temporal resolutions do affect the model’s performance, and the relationship between model performance and temporal resolution is not simply proportional.

**Table 2:** Evaluation results of multi temporal resolution

Timesteps	Kappa	Fuzzy Kappa
Timesteps 0 (No time data)	0.843	0.884
Timesteps 1 (Contains data from past year)	0.842	0.884
Timesteps 2 (Contains data from past two years)	0.871	0.948
Timesteps 3 (Contains data from past three years)	0.856	0.936

**Figure 5:**
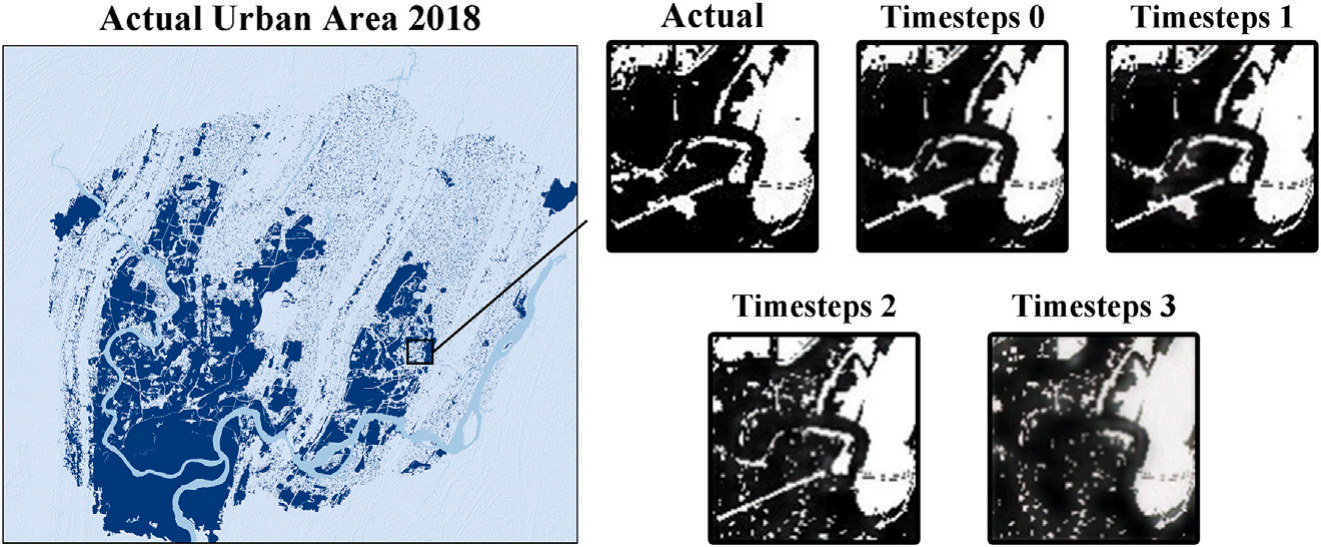
Simulation results of temporal models

Figure 5 shows that the urban areas simulated at a high temporal resolution differ significantly from the actual ones. Most of the fine urban areas such as villages are not simulated, and the details of the simulation also differ significantly from the actual situation. At timesteps 2 and higher temporal resolutions, this situation is well improved, and more details are accurately simulated by the model. However, at timesteps 3, inaccuracies such as missing simulations of some urban areas appear. When the timestep is higher, the model needs to learn longer term dependencies, which may lead to gradient disappearance or gradient explosion problems, thus affecting the training and performance of the model. In addition, the model may face the data sparsity problem, which can make it difficult for the model to accurately capture the key information in the sequence data, thus affecting the performance of the model. It is illustrated that a finer temporal resolution does not necessarily guarantee a better model performance, and the temporal resolution should be chosen based on the actual data situation.

### Model comparison with different spatial resolutions

For the prediction results with different spatial resolutions, we compared the urban cover in 2018 predicted by different models using different resolution training data with the real situation by Kappa coefficient, Fuzzy Kappa, and other parameters, and compared the SLEUTH, FLUS-Markov, and Markov-CA models for 30, 90, 180, 450, and 900 m resolution land use data. Following the model requirements, we provided land use data from 2009 to 2017 and utilized the same drivers, including road data, nighttime light data in 2010 and 2015, and population data in 2010 and 2015, to simulate each model’s urban area in 2018.

**Figure 6:**
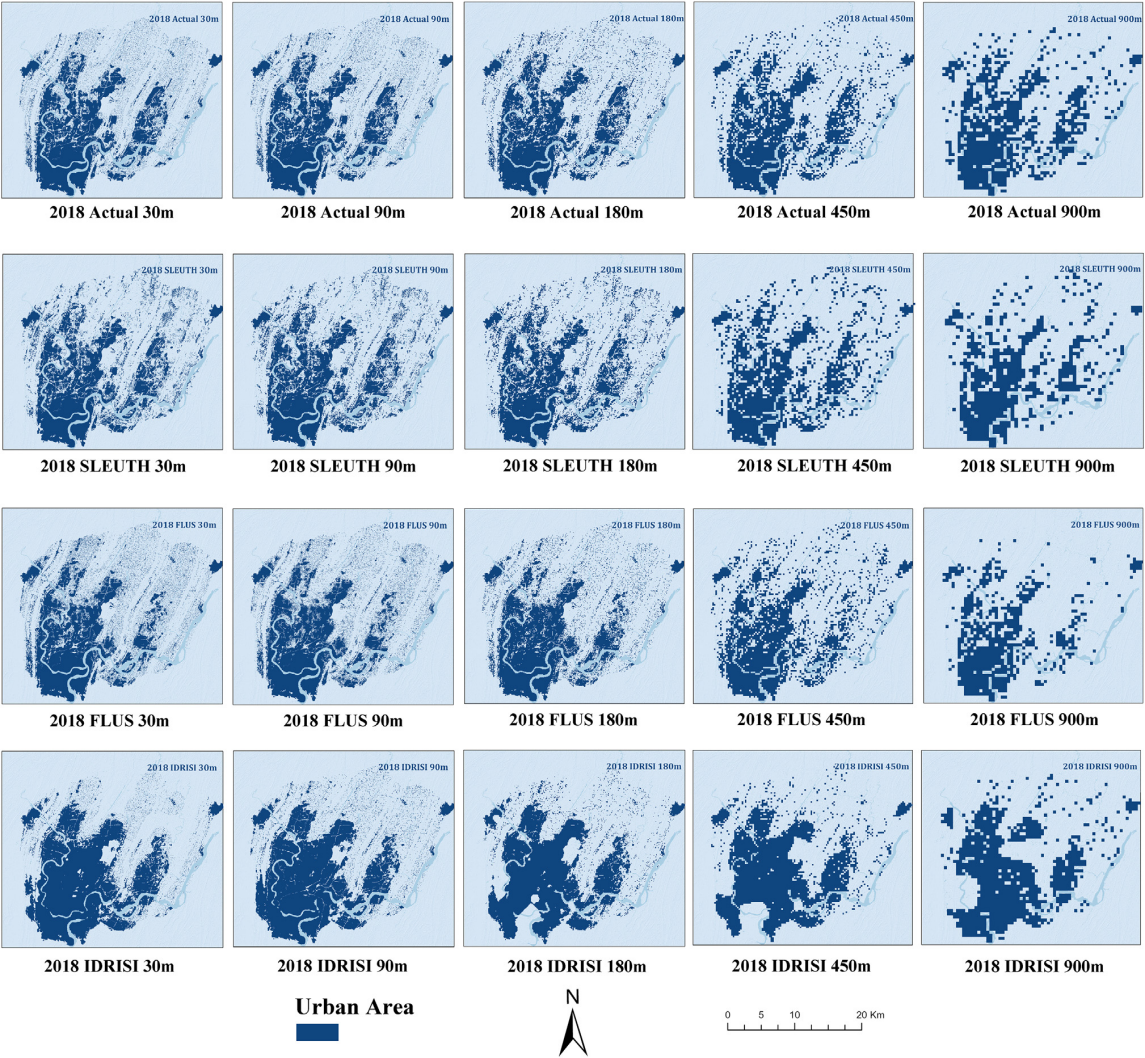
Simulated results of spatial models

The findings presented in Table 3 and Figure 6 suggest that the performance of all models in predicting urban expansion decreases as the resolution decreases. Among all the resolution data in this study, the FLUS model has the best performance for urban expansion prediction, followed by the SLEUTH model, and the IDRISI model performs the worst. The results indicate that the model’s performance for urban expansion simulation decreases with decreasing resolution and has a proportional relationship. However, if the training data had a resolution coarser than 450 m, all models showed a significant decrease in predictive power. For instance, at 900 m resolution, the Kappa coefficient of the SLEUTH model decreased by 26.9% to 0.629, while the Kappa coefficient of the FLUS model decreased by 9.93% to 0.78, and the Kappa coefficient of the IDRISI model was 0.655.

**Table 3:** Evaluation results of multi-spatial resolution

Model	Resolution	Kappa	Fuzzy Kappa
SLEUTH	30 m	0.864	0.876
	90 m	0.863	0.884
	180 m	0.862	0.888
	450 m	0.86	0.894
	900 m	0.629	0.771
FLUS	30 m	0.867	0.918
	90 m	0.872	0.932
	180 m	0.869	0.93
	450 m	0.866	0.928
	900 m	0.78	0.854
IDRISI	30 m	0.815	0.825
	90 m	0.72	0.781
	180 m	0.653	0.728
	450 m	0.644	0.746
	900 m	0.655	0.771

However, at 900 m resolution, the SLEUTH model loses its advantage for urban expansion simulation as it cannot learn effective spatial information due to the low resolution and the influence of the calibration process factor. On the other hand, the FLUS model’s ability to simulate urban expansion is better, and its overall simulation of urban areas is more accurate, ensuring its ability to predict urban areas is still more accurate at low resolutions. The prediction performance of the IDRISI model is the worst at low resolutions, and its prediction of urban areas is subject to errors. Its ability to predict urban area details is not as good as the other two models. The Kappa coefficients are all between 0.65 and 0.72 on the experimental data below 30 m resolution, and the simulation results have partial deviation from the real area. Furthermore, the Fuzzy Kappa coefficients at 30 m resolution are lower than those at coarser resolutions of 90, 180, and 450 m. At 30 m resolution, the Fuzzy Kappa for the SLEUTH model is 0.876, and the Fuzzy Kappa for the FLUS model is 0.918. One possible explanation for these values is that when the resolution is higher, the differences between the samples become smaller, leading to more errors in the classifier’s prediction. Therefore, a higher resolution dataset may result in a decrease in the classifier’s performance, which, in turn, leads to a decrease in the Fuzzy Kappa value. Conversely, when the resolution is lower, the prediction results of the classifier may be coarser, leading to a higher Fuzzy Kappa value.

### Spatiotemporal model result

**Figure 7:**
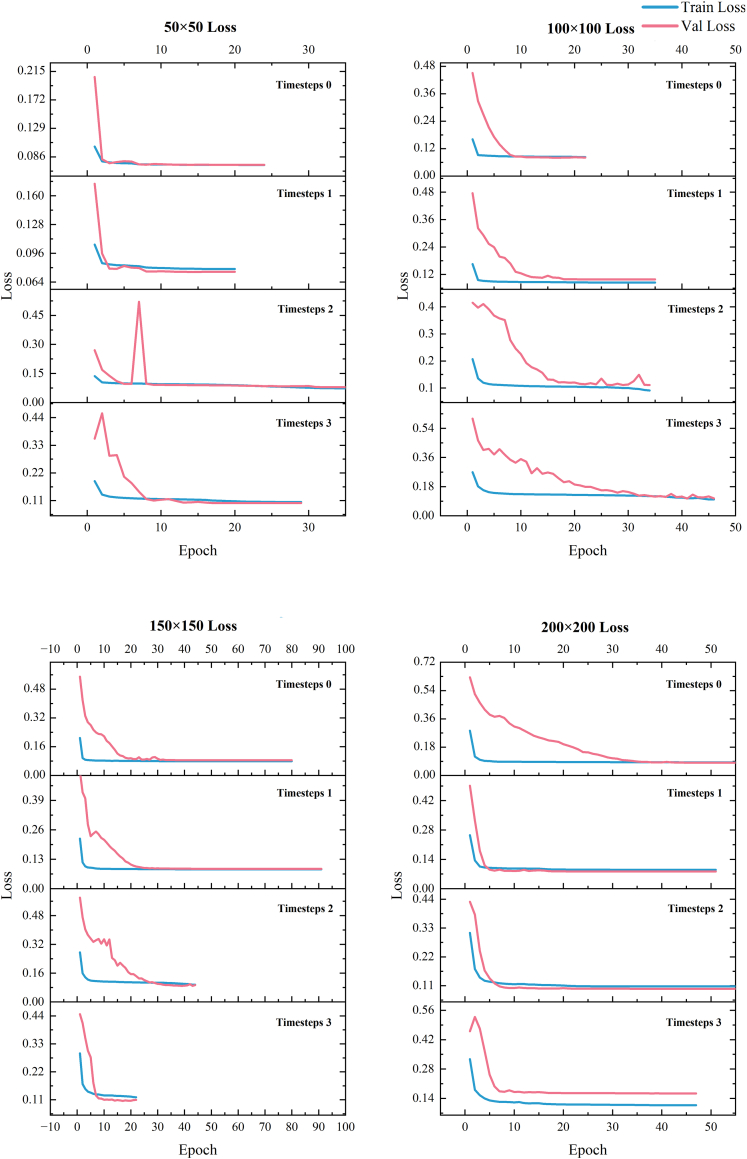
Spatial-temporal model training loss value curve

We segmented the original images into 50×50, 100×100, 150×150, 200×200 to include different spatial information into the model for training, and we processed each training data containing different spatial information using sliding window technique to include different temporal resolution information. The training results of the model are shown in Figure 7.

**Figure 8:**
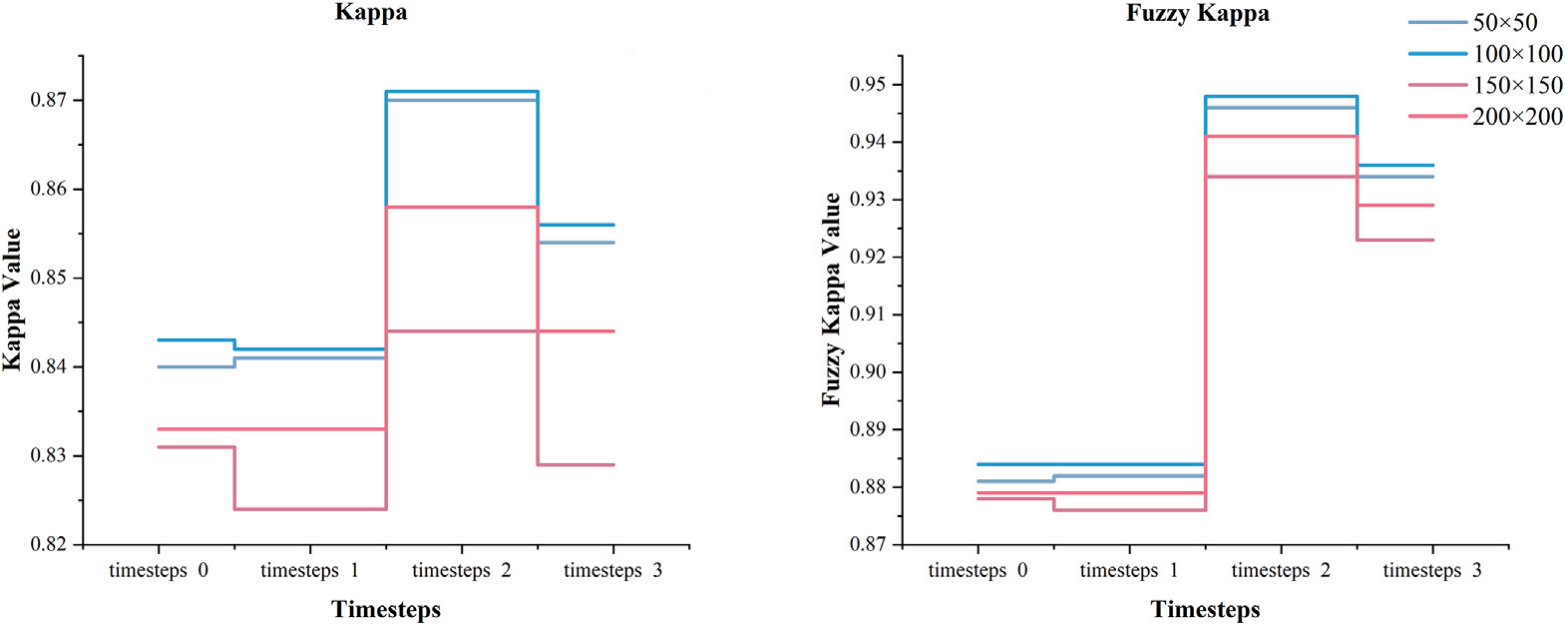
Kappa value comparison

The training of all models achieved a lower loss, indicating that the models gradually learned more data features and patterns during the training process, and the prediction results of the models were more accurate, and there were no overfitting and underfitting cases. We still take Kappa and Fuzzy Kappa (Figure 8) to evaluate the performance of the model in predicting the urban expansion process. From the data, the performance of the model shows a large gap between 50 × 50, 100 × 100 and 150 × 150, 200 × 200. The optimal Kappa coefficient is 0.87 and Fuzzy Kappa is 0.946 when 50 × 50 spatial information is included, and the optimal Kappa coefficient is 0.871 and Fuzzy Kappa is 0.948 when 100 × 100 spatial information is used for simulation. However, the more spatial information is included, the better the model performs. When the input features have a higher spatial resolution, the model needs to process more parameters and the model performance may suffer. When the model focuses too much on the spatial information, it may pay too much attention to the details of each pixel and ignore the overall structure and pattern. The optimal Kappa coefficient is 0.844 and Fuzzy Kappa is 0.934 under the inclusion of 150 × 150 spatial information, and the optimal Kappa coefficient is 0.858 and Fuzzy Kappa is 0.941 under the inclusion of 200 × 200 spatial information.

As can be seen from Table 4, the model performance is divided into two classes, one containing spatial information less than 100 × 100 and another containing spatial information less than 200 × 200. The more spatial information is included in each level, the better the model prediction is. Also, the model with different spatial information is always the best for the model with the past two years of time information (timesteps 2).

**Table 4:** Evaluation results of multi-spatiotemporal resolution

Spatial Extent	Timesteps	Kappa	Fuzzy Kappa
50 × 50	0	0.84	0.881
	1	0.841	0.882
	2	0.87	0.946
	3	0.854	0.934
100 × 100	0	0.843	0.884
	1	0.842	0.884
	2	0.871	0.948
	3	0.856	0.936
150 × 150	0	0.831	0.878
	1	0.824	0.876
	2	0.844	0.934
	3	0.829	0.923
200 × 200	0	0.833	0.879
	1	0.833	0.879
	2	0.858	0.941
	3	0.844	0.929

**Figure 9:**
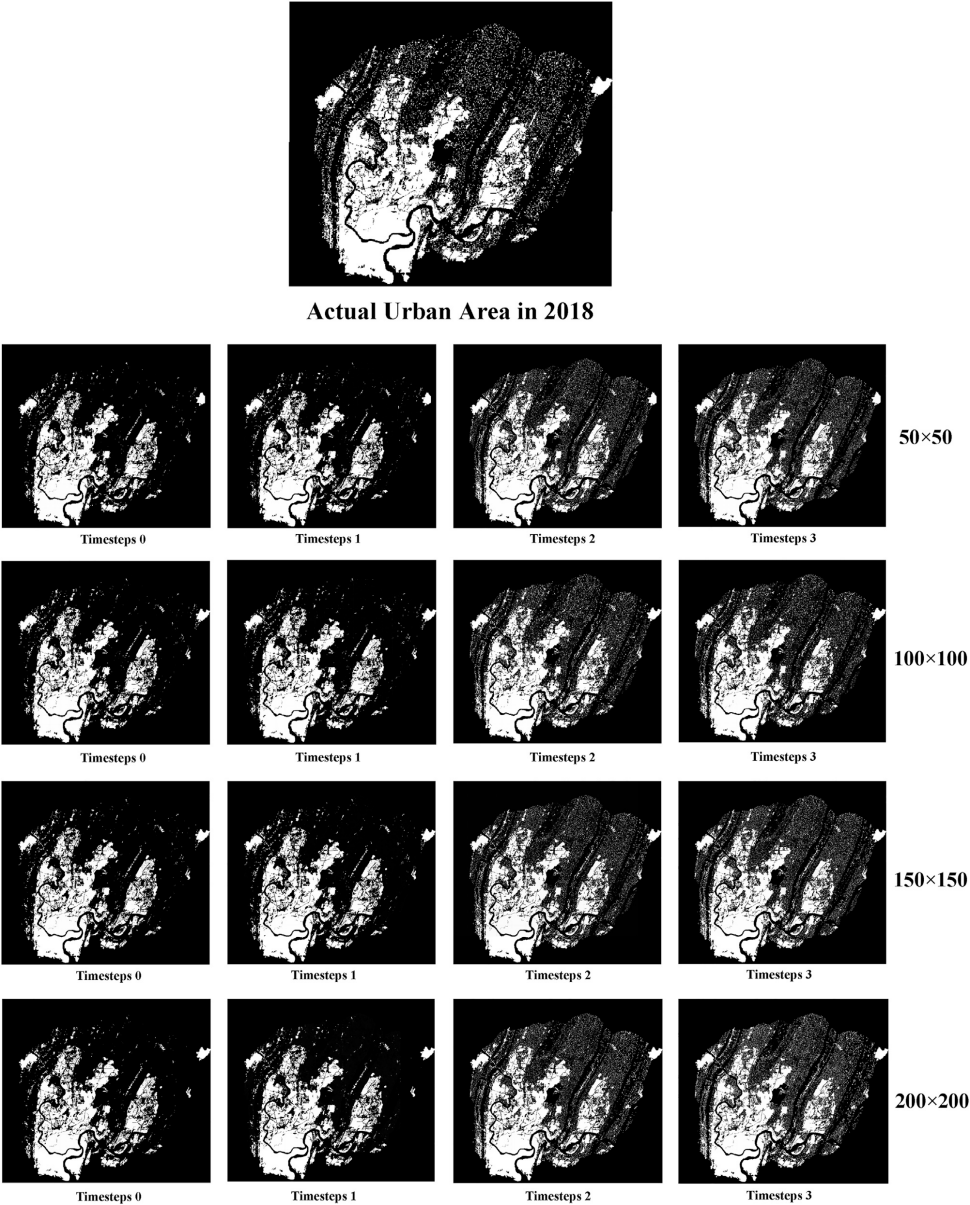
Simulation results of spatiotemporal models

**Figure 10:**
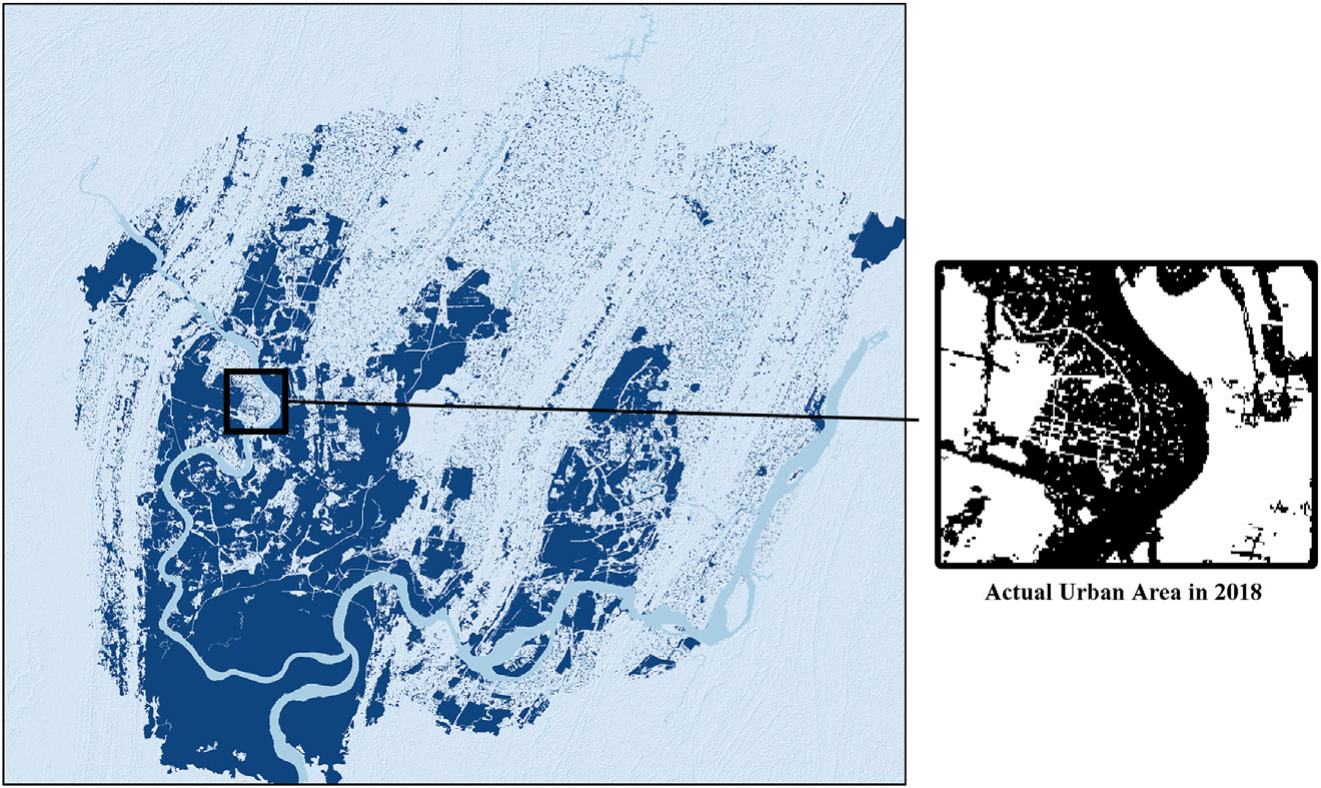
Selected zone to show the results

**Figure 11:**
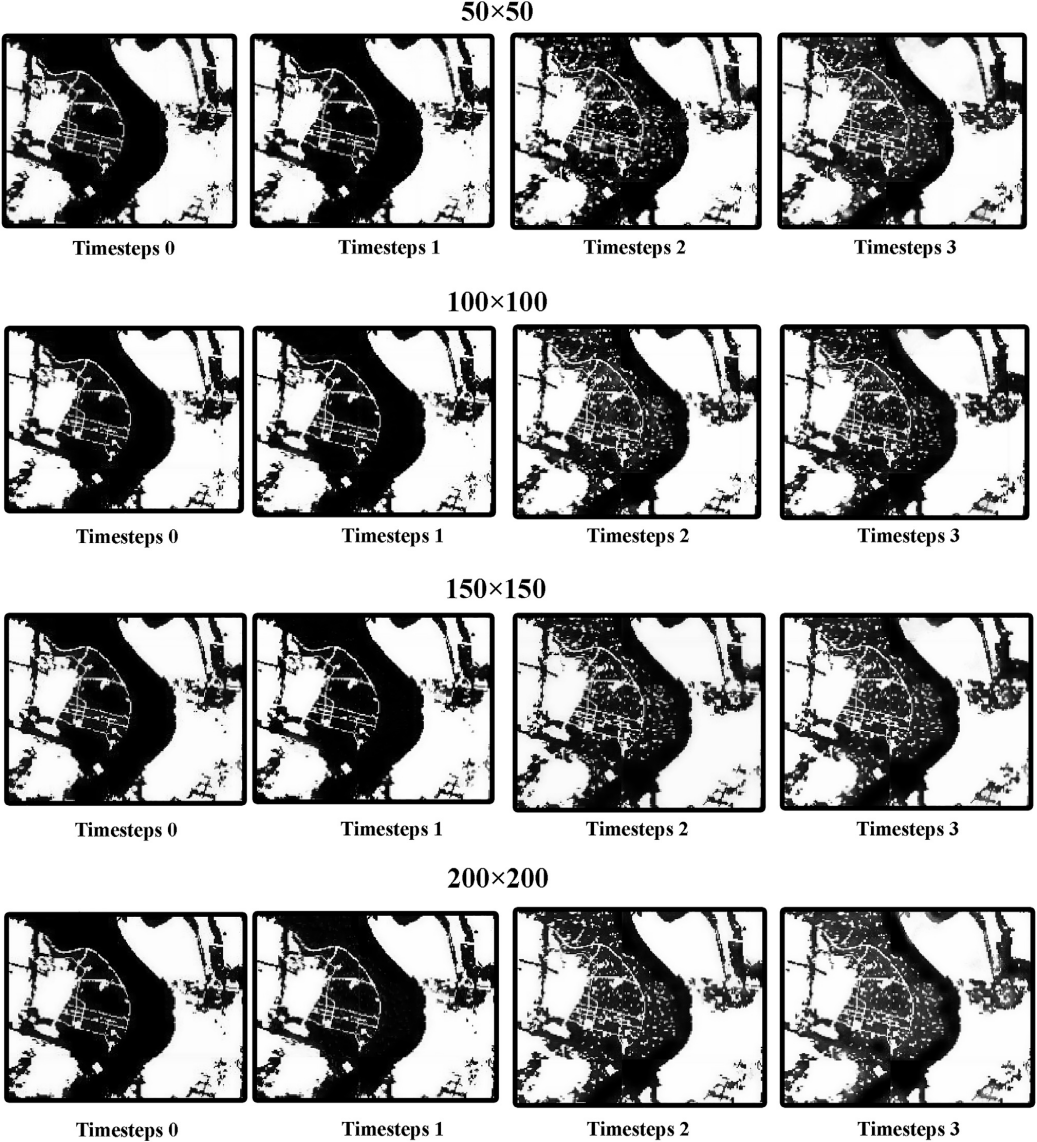
Details of the results in selected zone

The model with more spatial information is appears more generalizable visually (Figure 9), but there is a limit to this, and it does not necessarily mean that the inclusion of more spatial information will result in a more accurate model. The inclusion of more spatial information in the training data may cause the model to classify non-urban areas as urban areas, leading to a decrease in prediction performance. Figures 10 and 11 show that with the inclusion of more spatial information of higher temporal resolution, the model increasingly identifies non-urban areas as urban, and the inclusion of spatial information that is too high or too low can cause the model to lack information on details. Based on the joint analysis of simulated urban areas and their spatial distribution, the model parameters that deliver the best prediction performance are 100 × 100 with timesteps 2.

## Discussion

### The effect of long time series model in urban expansion simulation

The effect of long time series model in urban expansion simulation is important.^22^ Urban expansion is a long-term evolutionary process that requires consideration of urban planning and construction over multiple time periods. The long time series model can predict and simulate the urban development trend,^35^ which can provide a scientific basis for urban planning and construction. And from the experimental results, the optimal prediction results are obtained by a model with long time series data processing capability like convolutional LSTM. The long time series model can use historical data and trends to predict the direction and scale of future city development. For example, population growth trends and population migration patterns of future cities can be predicted by the model, thus providing a scientific basis for urban planning and construction. In addition, it can also predict future trends in economic, transportation, and environmental factors to help urban planners better formulate urban development strategies and policies. In addition, the model can optimize and adjust the urban expansion simulation process. For example, the model can be used to study the impact of urban expansion on the environment and resources,^36^ so as to develop reasonable urban planning and construction plans. During the urban expansion simulation process, the model can also help urban planners predict the demand and expansion direction of transportation and communication infrastructure within and around the city, so as to better meet the needs of urban development.

### Temporal resolution and spatial resolution effect on urban expansion simulation

Temporal resolution and spatial resolution are one of the key factors affecting urban expansion simulation. The process of urban development is a long-term evolutionary process that requires consideration of urban planning and construction over different time periods and future development trends.^37^ Urban expansion and development are a dynamic process that needs to consider the spatial relationships within the city and the surrounding areas.^38^ Different temporal and spatial information will bring different prediction results to the model. Different spatial resolutions will bring different prediction accuracies to different models, but the consistent result is too low that spatial resolution will significantly affect the prediction performance of the model, leading to large deviations in model predictions and even large prediction errors. However, a higher spatial resolution does not mean a higher model prediction accuracy. Excessive resolution means that the model needs to handle more details and data, which may lead to model overfitting, thus lowering the prediction accuracy.^39^ For the selection of spatial resolution, it is necessary to consider the complexity of the model and the limitation of computational resources to choose the most suitable spatial resolution for the model so as to achieve the optimal prediction results.

However, for the validation of different temporal resolutions, the experimental results show that the urban expansion simulation with training data containing temporal information of the past two years works best. It is confirmed that including temporal data does improve the performance for urban expansion simulation model, as demonstrated by other researchers.^40^ And different temporal resolutions do have an impact and ignoring long-term dependence significantly affects the prediction accuracy of the model. Considering different temporal resolutions and different spatial resolutions, the appropriate spatial and temporal resolutions combination can bring the optimal prediction effect. The selection of spatial and temporal information needs to be considered in conjunction with the selection of the model and the actual situation of the study area to optimize spatial and temporal resolution for the training of the urban expansion model.

### A spatial-temporal resolution optimization process in urban expansion model

In order to make the selection of the training data resolution for the urban expansion model more scientific and process oriented, and to achieve better accuracy of the urban expansion simulation, we designed a training data spatial and temporal resolution optimization process for the urban expansion model. Through our experiments, we found that the more temporal and spatial information is included, the better the model simulation will be. The reason for this result is that too much information may lead to overfitting of the model, where the model overfits the noise and details in the training data and ignores the general trends and patterns. In addition, too much information input may also increase the complexity of the model, making it more difficult to train and interpret, and thus leading to reduced results. The model has the highest accuracy for urban expansion simulation when the training data contain a specific range of spatiotemporal information (i.e., a specific spatiotemporal resolution), which needs to be decided by an optimization process.

Based on the results, we design a spatial-temporal resolution optimization process for training data. (1) Based on the actual data situation, we select data containing the past 1–3 years as the training dataset. For spatial resolution, certain experiments and analyses are needed to find the best balance between spatial resolution and model accuracy. If convolutional neural networks are used for urban expansion prediction, it is recommended to include spatial information below 150×150. If a traditional model is used for prediction, it is recommended to select data with a resolution of 90–450 m for model training. (2) Pre-process and clean the training data and generate long-term time data. These operations can help the model understand the data better and improve the training effect and generalization ability of the model. (3) Select a suitable model and algorithm for training. (4) Evaluate the training results by Kappa coefficients and other parameters, and according to the evaluation results, the model and the spatiotemporal resolution of training data are optimized again.

Moreover, we must point out that the research scale in this study is medium scale, i.e., the city is the main object of the study. However, the optimal spatial and temporal resolution are scale dependent, and will likely vary at different simulation scales, and the results produced must be different at the global scale and the neighborhood scale. The selection of the optimal resolution requires specific experiments and analysis according to the study scale, which is our future research direction.

### Conclusions

In recent years, urban expansion simulation has become a significant research area due to the rapid expansion of cities. In this paper, we investigated the accuracy of different spatial and temporal resolutions and different urban expansion models for urban expansion simulation. We propose a new process to optimize the spatial and temporal resolution of urban expansion simulation models and determine the most suitable range of temporal and spatial resolutions to obtain optimal urban expansion simulation results. Our findings indicate that the long time series model effectively handles the simulation of urban expansion and accurately predicts the development process of a district with better accuracy than traditional urban expansion prediction models and good robustness.

### Limitations of the study

However, our analysis only considers the effect of spatial-temporal resolution on the simulation of urban expansion and does not account for the indirect effect of each influencing factor on urban areas. These factors, such as consumption and employment, are challenging to quantify and add to a long-time network model. Additionally, consumption patterns in cities differ significantly from those in rural areas, posing a challenge to our model. Although it is beyond the scope of this paper to comprehensively assess the impact of each factor on urban expansion simulation, these are influential factors that cannot be ignored in urban expansion simulation models. Therefore, the effects of these factors on the model are direct or indirect variables that need to be considered.

## STAR★Methods

### Key resources table

**Table:** 

REAGENT or RESOURCE	SOURCE	IDENTIFIER
**Urban relating data**		

Land-use raster	Resource and Environment Science and Data Center, China	https://www.resdc.cn/DOI/DOI.aspx?DOIid=54
DEM		https://www.resdc.cn/data.aspx?DATAID=217
Population density		https://www.resdc.cn/DOI/DOI.aspx?DOIID=32
Urban Roads	Open Street Map	https://www.openstreetmap.org/
Nighttime Light	NOAA	https://ngdc.noaa.gov/eog/dmsp/download_radcal.html
Urban Gary Image	Chongqing Public Service Platform of Geographic Information	http://t0.tianditu.gov.cn/img_w/

**Software and algorithms**		

ArcGIS Pro 2.5.2	ESRI	https://www.esri.com/en-us/arcgis/products/index?rmedium=esri_com_redirects01&rsource=/en-us/arcgis/products
Tensorflow	Python Society	https://www.tensorflow.org/api_docs/python/tf

### Resources availability

#### Lead contact

Further information and requests for resources should be directed to the lead contact, Dr. Tingting Xu, xutt@cqupt.edu.cn.

#### Materials availability

This study did not generate new unique reagents.

#### Data and code availability

• The data produced on this study can be downloaded (publicly accessible) via Github: https://github.com/VladimirSu/Resolution-effect-on-fast-growing-urban.
• The original codes of this paper can be downloaded (publicly accessible) via Github: https://github.com/VladimirSu/Resolution-effect-on-fast-growing-urban.
• For any additional information required to reanalyze the data reported in this paper, please contact the lead author.
• Resolution-effect-on-fast-growing-urban
• Contact: 2020214785@stu.cqupt.edu.cn
• Hardware requirements: CPU: Intel(R) Xeon(R) Gold 6130 CPU @ 2.10GHz
• GPU: NVIDIA V100-32 GB Memory: 25GB
• Program language: Python 2.7 Software required: Jupyter Notebook;
• Program size: 24 kB

### Experiment model and subject details

This study does not use experimental models.

### Method details

#### Research framework

The simulation process involves three primary steps (Method S1). The first step is to examine the impact of temporal resolution on the predictive performance of urban expansion using the ConvLSTM model. To accomplish this, the 2009–2018 data were divided into different time steps using the slide window technique, and the resulting multi-temporal information was saved into the training data. The second step involves exploring the effect of spatial resolution (30 m, 90 m, 180 m, 450 m, 900 m) on the performance of the SLEUTH, FLUS, and IDRISI models. The third step involves determining the impact of different spatiotemporal information on urban expansion simulation performance. The trained ConvLSTM model was utilized to predict the urban area in 2018. Finally, the performance of different models in simulating urban expansion was evaluated using the Kappa coefficient and Fuzzy Kappa to find out the best combination of sptio-temporal resolution input.

#### Data pre-processing

ConvLSTM model was fed by urban areas extracted from 30 m resolution land use data and divided into different blocks at a size of 100 × 100, which contains different spatial information (Method S2). After processing, we obtained image data for each subregion from 2009 to 2018. To train the ConvLSTM, the input data will have a size of, where w is image block’s width, h is image block’s height. The output size is also. After stitching all the blocks together, a map of urban areas simulated by ConvLSTM in 2018 will be produced.

To include temporal information data with different time steps, we used the time sliding window technique (Method S3). It is a mechanism for pre-processing sequential data. In LSTM, sliding window input is usually used to process temporal data. The size of the sliding window is usually adjustable, and once the size of the sliding window is determined, the temporal data can be divided into several sliding windows. Each sliding window is a subsequence with a fixed size, so that the whole sequential data can be decomposed into several short sequences. For each sliding window, the ConvLSTM can process and obtain the corresponding output. With the sliding window mechanism, the ConvLSTM can process temporal data of any length and extract useful information from it.

#### Spatial model

The SLEUTH, IDRISI, and FLUS, which are the three most popular urban simulation models,^7^^,^^26^^,^^27^^,^^28^^,^^29^ have been used to test how the spatial resolution of the input variables could affect the results. The differences between these models are shown in Method S6.

#### Temporal model

The LSTM model, known as Long Short-Term Memory, is a recurrent neural network (RNN) for processing sequential data, which has stronger memory capability and can capture temporal correlation in long sequence data.^30^^,^^31^^,^^32^ The basic components of the LSTM model includes input gates, forgetting gates and output gates. The input and forgetting gates control the input and forgetting of information, while the output gate generates output based on the current state and input information (Method S4).

Specifically, the input of the LSTM consists at the current moment and the state at the previous moment. For input processing, the LSTM controls what information can enter the current state through the input gate, and then calculates the new state based on the current state and the input information. Meanwhile, the forgetting gate controls the kind of information that can be forgotten to prevent the earlier information from interfering with the later computation. Finally, the output gate produces the output based on the current state and the input information. The gating mechanism allows the model to adjust the information flow between different time scales, thus allowing better processing of information at different time scales.

To investigate the effect of different temporal resolutions on the performance of the model for urban expansion simulation, we constructed a prediction architecture consisting of three convolutional LSTM layers (Method S5) with three convolutional kernel sizes of 7 × 7, 5 × 5, 3 × 3, two Batch Normalization (BN) layers and one convolutional layer for the output prediction image.^33^ The input and output dimensions of the model are (100, 100, 3). With the sliding window technique, we can create datasets containing different temporal data for training prediction networks.

The calculation process can be summarized as the following mathematical equation.

(Equation 1) $$I_{ij}^{year}=\left(\begin{array}{cc} B_{11} & B_{1n}\\ B_{n1} & B_{nn} \end{array}\right)\left(2009\leq year\leq 2017\right)$$

where *B* denotes the sub-images split into different block sizes, i,j denotes the area number of the sub-image located in the original image (row *i* and column *j*), n denotes the number of sub-images into which the original image is split. The year represents the time of the urban area shown in the original image.

(Equation 2) $$x_{ij}^{t}=\left[I_{ij}^{year}\;I_{ij}^{year+t}\right]\left(t=0,1,2,3\right)\left(2009\leq year\leq 2012\right)$$

(Equation 3) $$y_{ij}^{t}=\left[I_{ij}^{year+t}\;I_{ij}^{year+2t}\right]\left(t=0,1,2,3\right)\left(2013\leq year\leq 2017\right)$$

(Equation 4) $$X_{t}=\left(\begin{array}{cc} x_{11}^{t} & x_{1n}^{t}\\ x_{n1}^{t} & x_{nn}^{t} \end{array}\right)\left(t=0,1,2,3\right)$$

(Equation 5) $$Y_{t}=\left(\begin{array}{cc} y_{11}^{t} & y_{1n}^{t}\\ y_{n1}^{t} & y_{nn}^{t} \end{array}\right)\left(t=0,1,2,3\right)$$

Where t represents timesteps, $T_{ij}^{t}$ represents sub-image in row *i* and column *j* with timesteps t. $X_{t},Y_{t}$ represents the input data to train the model.

#### Spatial-temporal model

Convolutional LSTM (ConvLSTM) is a neural network structure that combines convolutional neural network (CNN) and long-short term memory network (LSTM). It has a good performance in processing time series data. The core idea of ConvLSTM is to replace the memory unit of LSTM with a convolutional layer, so that information can be processed and delivered in both temporal and spatial dimensions. Also, ConvLSTM can improve the performance of the model by stacking multiple ConvLSTM layers.

The updating calculation formula for the *t*-th layer of ConvLSTM is expressed as follows:

(Equation 6) $$\left\{ \begin{array}{c} i_{t}=\sigma \left(W_{xi}*x_{t}+W_{hi}*H_{t-1}+W_{ci}\circ C_{t-1}+b_{i}\right)\\ f_{t}=\sigma \left(W_{xf}*x_{t}+W_{hf}*H_{t-1}+W_{cf}\circ C_{t-1}+b_{f}\right)\\ o_{t}=\sigma \left(W_{xo}*x_{t}+W_{ho}*H_{t-1}+W_{co}\circ C_{t-1}+b_{o}\right)\\ C_{t}=f_{t}\circ C_{t-1}+i_{t}\circ \tanh\left(W_{xc}*x_{t}+W_{hc}*H_{t-1}+b_{c}\right)\\ H_{t}=o_{t}\circ \tanh\left(C_{t}\right) \end{array}\right.$$

Where $i_{t}$ represents input gate, σ represents sigmoid layer and will decide what value need to be updated. $f_{t}$ represents forget gate, sigmoid layer will realize a nonlinear mapping to remember what is important and forget what is irrelevant. A tanh layer creates a new vector of candidate values that will be added to the state. Then, $c_{t-1}$ will be updated as $c_{t}$. Multiply the old state with $f_{t}$, discard the information that needs to be discarded. This is the new candidate value, which changes depending on how much we decide to update each state. $W_{xi},W_{hi},W_{ci},W_{xf},W_{hf},W_{cf},W_{xo},W_{ho},W_{co}$ are the 2D convolutional kernel. The output gate controls $o_{t}$.

Similarly, after exploring the effect of different temporal resolutions on the model prediction performance under the same spatial information through the ConvLSTM model, we added different spatial information to the variables to explore how the combined effect of different spatial and temporal resolutions affect the model’s performance in simulating urban expansion. We used the same prediction architecture as the temporal model to predict the urban coverage of the Liangjiang New District in 2018 by inputting different data which contain multi-spatial information: divide the urban area image to n image blocks of 50×50, 100×100, 150×150, 200×200 . The hyperparameters we used in model is shown as Method S7.

#### Model validation

In this study, both Kappa and fuzzy Kappa were used to validation the model outputs. The Kappa coefficient is a metric used to assess the quality of classification, and it measures the agreement between the predicted and actual results. We calculated the kappa coefficients from the simulated 2018 urban expansion at different spatial resolutions of SLEUTH, FLUS, and CA-Markov to the actual 2018 urban expansion and compared them to explore the impact on the model prediction performance at different spatial resolutions.

Fuzzy Kappa serves as a statistical measure employed to assess the precision of classification models, which can take into account the uncertainty and ambiguity of the results. Compared with the traditional Kappa coefficients, Fuzzy Kappa assigns a weight coefficient to each of these three parts and calculates the weighted average to get the final evaluation result, which can better handle multi-category and fuzzy classifications.^34^

### Quantification and statistical analysis

The results of the accuracy evaluation are shown in Table 4, and the inclusion of different temporal information under the 100 × 100 spatial information range does lead to differences in the results. Under timesteps0 and timesteps1, the predictive performance of the models is similar, with Kappa coefficients of 0.843 and 0.842, respectively, and the Fuzzy Kappa is 0.884 for both. However, the predictive performance of the models trained by including temporal data of the past two and three years is greatly improved, with the Kappa coefficient for timesteps2 of 0.871, which is higher than timesteps0 (0.843) and timesteps1 (0.842), and the Fuzzy Kappa is 0.948, which is higher than timesteps0 and timestep1 (0.884). It indicates that higher temporal resolution does improve the prediction performance of the model, and temporal resolution is a critical factor that cannot be ignored in urban expansion research. For the prediction results of different spatial resolutions, we compared the urban coverage 2018 predicted by different models using different resolutions of training data with the actual situation through parameters such as Kappa coefficient, Fuzzy Kappa, etc. We compared the urban expansion simulation results of SLEUTH, FLUS-Markov, and Markov-CA models. From the results, the predictive performance of all models weakened with the decrease in resolution. However, a significant decrease in the predictive ability of all models was observed with training data below 450 m resolution. Having verified that both temporal and spatial resolutions have a particular effect on the model’s performance in simulating urban sprawl, in order to explore the effect of combining different temporal and spatial resolutions on the model, we segmented the original image into 50 × 50, 100 × 100, 150 × 150, and 200 × 200 to include different spatial information in the model for training. The optimal Kappa coefficient is 0.87, and the Fuzzy Kappa is 0.946 for 50 × 50 spatial information. The optimal Kappa coefficient is 0.871, and the Fuzzy Kappa is 0.948 for 100 × 100 spatial information, which verifies that the model performance can be improved by including more spatial information in the training data.
