# Supplementary material for: Influence of multiple spatiotemporal resolutions on the performance of urban growth simulation models
Source: iScience. 2023 Nov 30;27(1):108540. doi: 10.1016/j.isci.2023.108540 (PMC10755367; doi:10.1016/j.isci.2023.108540)
Supplement: Document S1. Methods S1–S7 [file mmc1.pdf]

iScience, Volume *27*

Supplemental information

Influence of multiple spatiotemporal resolutions on the performance of urban growth simulation models

Tingting Xu, Heng Su, Biao He, Aohua Tian, and Jianing Guo

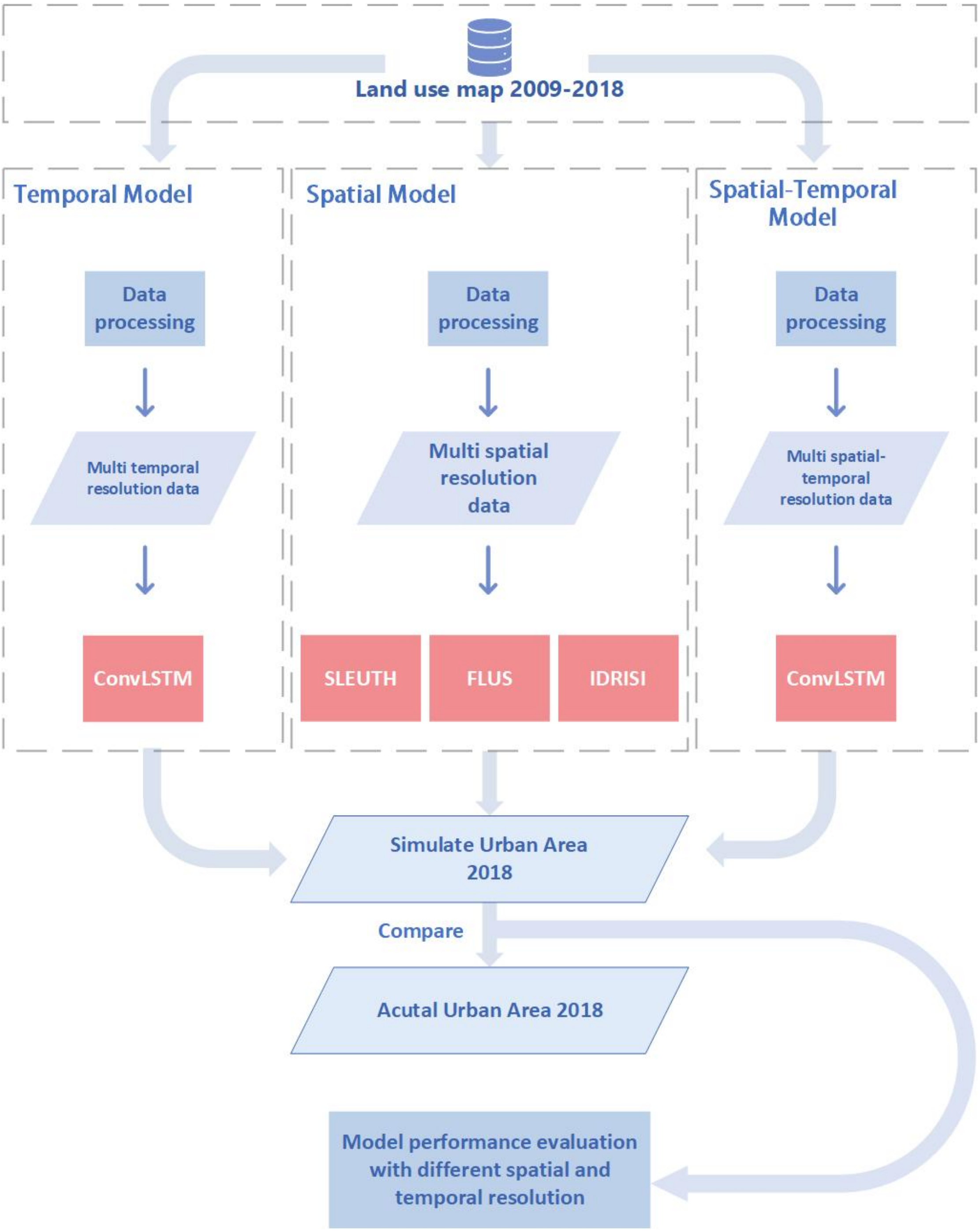

Methods S1: Research work flow chart

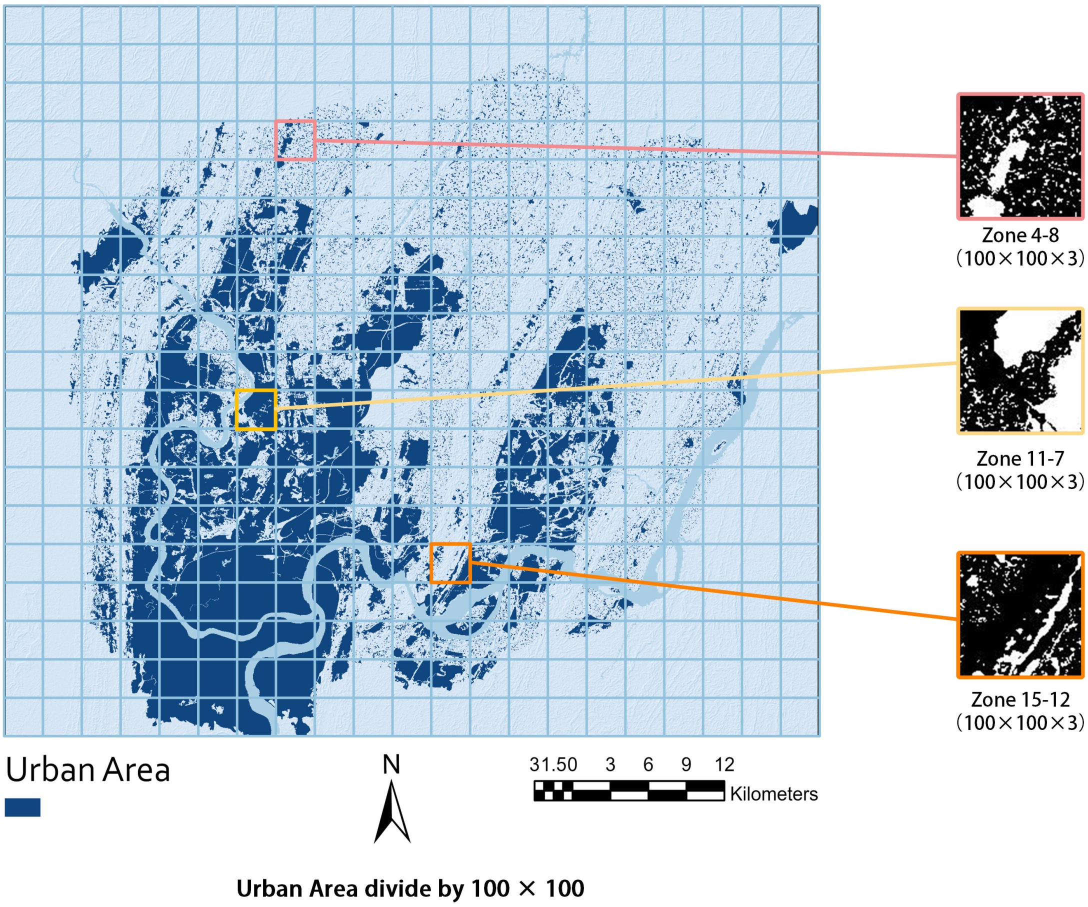

Methods S2: Training data process method

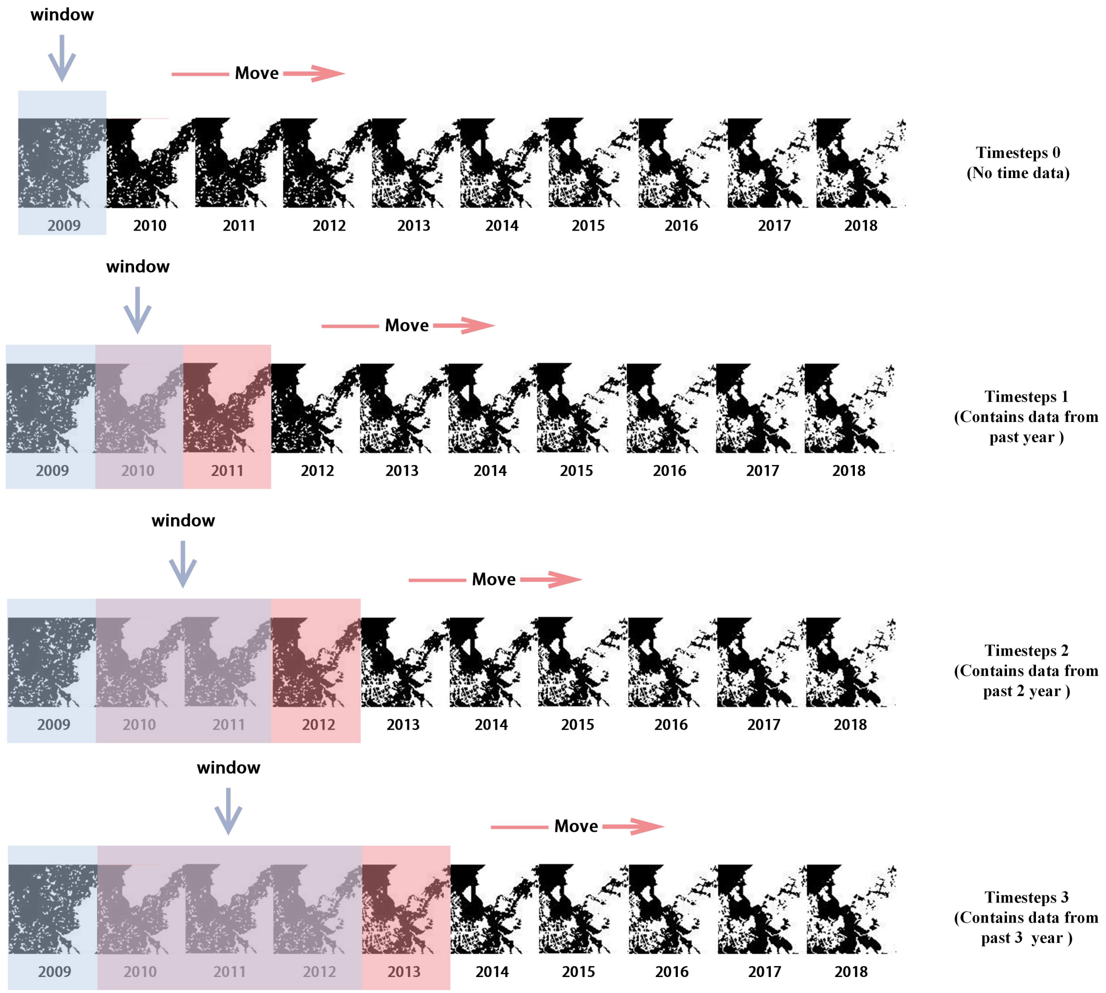

Methods S3: Slide window technique

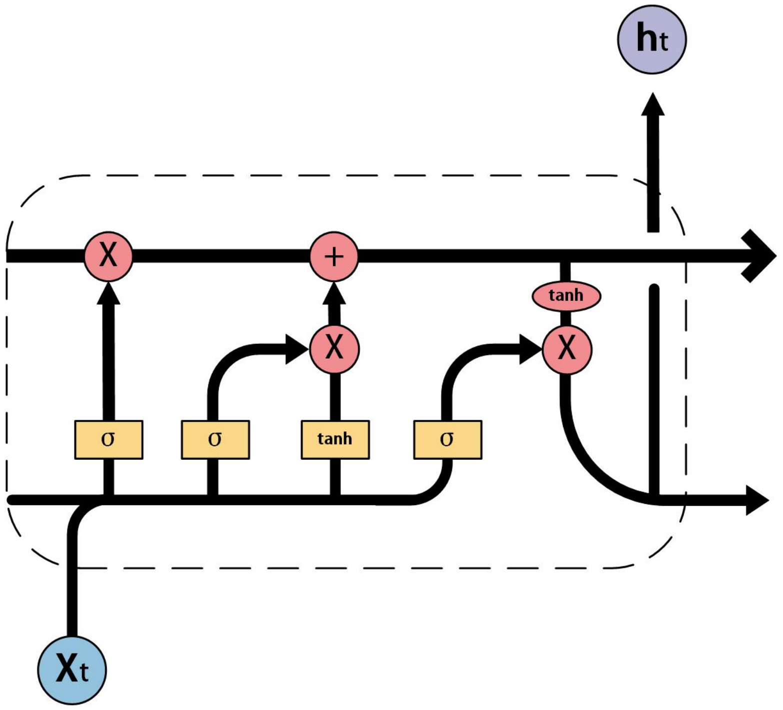

Methods S4: Network structure of LSTM

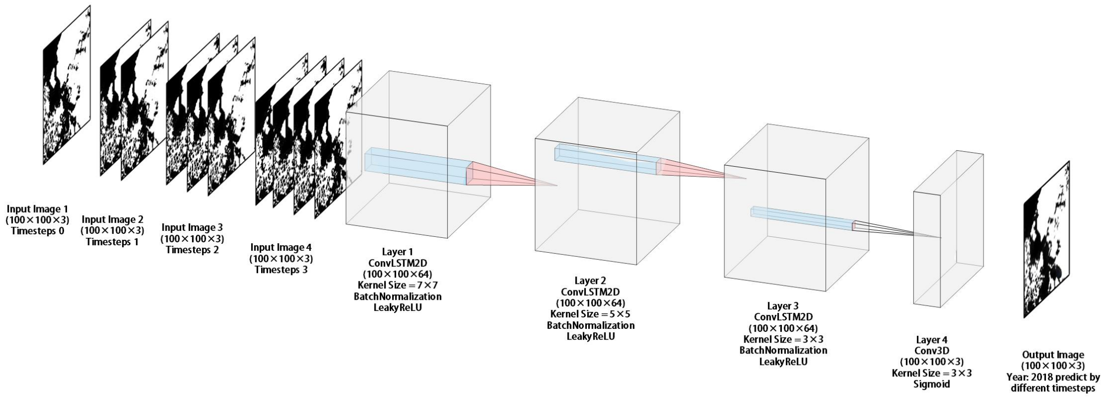

Methods S5: Spatial-temporal model structure

Tables of the STAR Method

Methods S6. Model Overview.

Model	FLUS	SLEUTH	IDRISI
Inputs Required	Driven Factors, Urban area, Land-use data	Slope, Land-use data, Exclusion, Urban, Transport, Hillshade	Land-use data, Transport, Urban area, Driving Factors
Advantages	Ability to consider a variety of factors, such as physical and demographic characteristics of the city, population growth, land use changes, transportation network development, etc., with high forecasting accuracy.	High prediction accuracy	High prediction accuracy and wide application range.
Disadvantages	Requires the use of cellular automata theory and genetic algorithms, requiring high mathematical and computer skills.	Fewer drivers, considering only the physical and demographic characteristics of the city, land use changes, transportation network development, etc.	The operational difficulty is high.
Distinctions	Based on cellular automata theory and genetic algorithm, it is able to consider more factors and has higher prediction accuracy.	Based on cellular automata theory, it is easy to operate but has fewer predictors.	Wide range of application based on remote sensing technology and GIS analysis methods

Methods S7. Hyperparameters of ConvLSTM model.

**Hyperparameters**	**Value**
Batch size	32: (50×50 timesteps 0-3, 100×100 timesteps 0-3 150×150 timesteps 0-2)<br>16: (150×150 timesteps 3, 200×200 timesteps 0-2)<br>8: (200×200 timesteps 3)
Optimizer	Adam
Loss Function	Binary cross-entropy
Hidden Layer	4
Activation Function	LeakyReLU (ConvLSTM)<br>Sigmoid (Conv3d)
Strides	1
Padding	same
